# Antibiofilm Efficacy of Luteolin Against Single and Dual Species of *Candida albicans* and *Enterococcus faecalis*

**DOI:** 10.3389/fmicb.2021.715156

**Published:** 2021-10-15

**Authors:** Yuting Fu, Wenjing Wang, Qiao Zeng, Ting Wang, Weidong Qian

**Affiliations:** School of Food and Biological Engineering, Shaanxi University of Science and Technology, Xi’an, China

**Keywords:** *Candida albicans*, *Enterococcus faecalis*, luteolin, mono- and dual-species biofilm, antibiofilm activity

## Abstract

*Candida albicans* and *Enterococcus faecalis* biofilm-associated infections have been a huge challenge to the medical community. However, the efficacy of natural products against mixed biofilms of *C. albicans* and *E. faecalis* still remains largely unexploited. The aim of this study was to evaluate the efficacy of luteolin against planktonic cell growth, adhesion, and biofilm formation of *C. albicans* and *E. faecalis* in single and mixed cultures *in vitro*. The results showed that the minimum inhibitory concentrations of luteolin against planktonic cells of *C. albicans*, *E. faecalis*, and mixed cultures were 32 and 64 μg ml^–1^, respectively. The results displayed that a remarkable variation in biofilm biomass, viability, structure, and composition of single and dual-species biofilms formed by mono- and dual-species biofilms of *C. albicans* and *E. faecalis* in the presence of luteolin was confirmed by mainly crystal violet staining assay (CVSA), optical microscope, field emission scanning electron microscope (FESEM), and confocal laser scanning microscope (CLSM). The tolerance of luteolin-treated single- and dual-species biofilms to antibiotics was found to obviously decrease, and the loss of biofilm matrix components (mainly polysaccharides and proteins) was revealed by CLSM. Moreover, luteolin was effective at inactivating biofilm cells, as well as destructing preformed biofilm structures by single and dual species by CVSA, FESEM, and CLSM. Collectively, these data indicate the potential of luteolin as a promising antibiofilm agent for the therapeutic management of biofilm-related infections induced by single and dual species of *C. albicans* and *E. faecalis*.

## Graphical Abstract

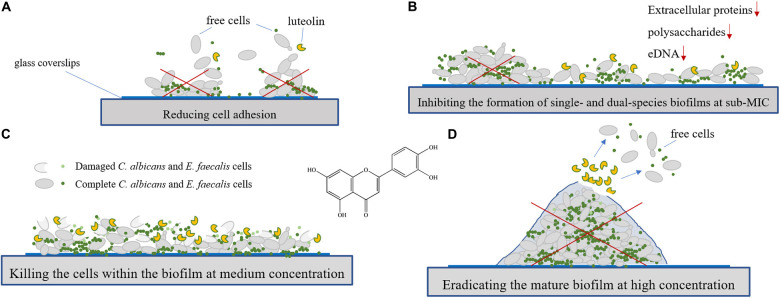

## HIGHLIGHTS

- Antibiofilm efficacy of luteolin against single and dual species of *C. albicans* SC5314 and *E. faecalis* 20033 was explored.
- Luteolin effectively inhibited the biofilm formation of *C. albicans* SC5314, *E. faecalis* 20033, and mixed cultures and killed *C. albicans* and *E. faecalis* biofilm cells.
- Luteolin could disperse efficiently preformed biofilms of *C. albicans* and *E. faecalis* single and dual species.

## Introduction

*Candida albicans* is the common fungi found as a normal commensal of human gastrointestinal flora. *C. albicans* has also been clinically found in oral niches, such as periodontal pockets and endodontic canals (Koo et al., 2018). *C. albicans* has been recognized as one of the most opportunistic fungal pathogens causing invasive infections under certain circumstances (Mane et al., 2012). Furthermore, the majority of *C. albicans* oral infections are closely related with its ability to produce biofilms (Tsui et al., 2016). Biofilm-growing *C. albicans* cells are particularly resistant to both antifungal drugs and host immune defenses compared with the planktonic counterparts (Wall et al., 2019). The oral cavity is colonized by a highly diverse microbial community of up to 700 different species of fungi and bacteria (Marsh and Zaura, 2017). In oral communities, there is increasing interest especially in the cross-kingdom interactions between fungi and bacteria, which form dense, surface-associated, socially organized communities called oral biofilms (Bowen et al., 2018). In the oral biofilm formed on mucosal surfaces, *C. albicans* is one of the most commonly detected fungal species (Rocha et al., 2018). Normally, within the host, mechanical removal of loosely adhered *C. albicans* by the physiological flushing effect of saliva and the peeling-off action of epithelial cells from mucosal surfaces are predominant factors in host defense against overgrowth of *C. albicans* biofilm in the soft tissues (Williams and Lewis, 2011). Moreover, oral colonization by *C. albicans* in mucosal sites is also hindered by the balanced commensal bacterial community in a healthy host, thereby inhibiting biofilm formation by *C. albicans* (Pereira-Cenci et al., 2008; Krishnamoorthy et al., 2020). However, recent studies have shown that certain bacterial species may overgrow as a result of the disturbed equilibrium between microbial habitats due to immunosuppressive conditions and thereby contribute to mutualistic relationships with *C. albicans* (Bertolini et al., 2019). Such mutualism has been implicated in the formation of a mixed biofilm between *C. albicans* and oral bacteria in both *in vitro* and mouse models (Xu et al., 2014; Bertolini et al., 2019). Furthermore, the mixed biofilms have been the major driver of the severity of dental caries and mucosal infections due to its increased resistance to antimicrobial therapies (Karygianni et al., 2020). *C. albicans* often forms polymicrobial biofilm communities through its association with a variety of bacterial pathogens at mucosal sites (Falsetta and Koo, 2014). For instance, representative streptococci from the mitis group, such as *Streptococcus mitis*, *Streptococcus oralis*, *Streptococcus sanguinis*, and *Streptococcus gordonii* form robust mixed biofilms when co-cultivated with *C. albicans* in different experimental models (Souza et al., 2020).

*Enterococcus faecalis*, a commensal gram-positive, facultative anaerobic bacterium, normally inhabits the gastrointestinal tract (Lee et al., 2019). Nonetheless, *E. faecalis* is also well-known to cause several infections ranging from wound infection to life-threatening infections, including endocarditis, bacteremia, and meningitis, and urinary tract infections (Zhang et al., 2015). Graham et al. (2017) demonstrated the inhibitory effect of bacteriocin from *E. faecalis* OG1RF on biofilm formation of *C. albicans* SC5314 through the inhibition of hyphal formation on abiotic surfaces. However, meanwhile, growing evidences show that hospitalized patients are predisposed to *E. faecalis* and *C. albicans* co-infections, such as tongue mucosal infections, sputum, sepsis, and root canal infections (Dahlen et al., 2012; Abusrewil et al., 2020). More recently, depletion of *E. faecalis* in mice treated with antibiotics impaired oral mucosal invasion of *C. albicans*, highlighting the necessity of the contribution of *E. faecalis* to dysbiosis due to *C. albicans* on oral mucosa of mice (Krishnamoorthy et al., 2020). Similar studies also indicate that the co-infection due to the cross-kingdom interaction between *C. albicans* and major bacteria may be positively associated with increased disease severity (Shirtliff et al., 2009; Kalpana et al., 2020). In particular, infections caused by *C. albicans*–bacteria mixed biofilms have also been implicated in higher mortality rates when compared to those due to a single species of microorganism (Morales and Hogan, 2010). Therefore, exploitation of novel agents to inhibit biofilm formation and promote the dispersal of mature biofilms may ultimately enhance the treatment outcome, but to the best of our knowledge, few studies about the efficacy of natural products against *E. faecalis* and *C. albicans* mixed biofilms have been reported.

Luteolin is an interesting naturally occurring flavonoid in view of its pharmacological potentials including anti-oxidant and anti-inflammatory activities, which occurs in peanut shells and in many kinds of plants, such as fruits, thyme, peppermint, parsley, celery, and green pepper (Wang et al., 2008; Luo et al., 2019). Recently, luteolin exhibited antimicrobial or antibiofilm activities against pathogens, such as *Staphylococcus aureus*, *Bacillus cereus*, *Salmonella infantis*, *Escherichia coli*, and *Candida tropicalis* (Skroza et al., 2019; de Freitas et al., 2020). However, the efficacy of luteolin on single- and dual-species biofilms of *C. albicans* and *E. faecalis* has not yet been explored. The aim of this study was to evaluate the ability of luteolin to inhibit and disperse biofilms of single and dual species of *C. albicans* and *E. faecalis in vitro*.

## Materials and Methods

### Reagents

Luteolin (CAS: 491-70-3, high performance liquid chromatography purity ≥ 98%) was obtained from Chengdu Pulis Biological Science and Technology Co., Ltd. (Chengdu, China). Luteolin was dissolved in dimethyl sulfoxide (DMSO, Sigma-Aldrich) to obtain a final concentration of 20 mg ml^–1^ and diluted prior to use. Phosphate-buffered saline (PBS, pH 7.4) was purchased from Tianjin Tianli Chemical Reagent Co., Ltd. (Tianjin, China). Tryptic soy agar (TSA), tryptic soy broth (TSB), and yeast extract–peptone–dextrose (YPD) used in antimicrobial or antibiofilm activity tests were purchased from Difco Laboratories, Detroit, MI, United States. Gatifloxacin was purchased from Beijing Solarbio Science and Technology Co., Ltd. (Beijing, China). Film tracer SYPRO Ruby (SYPRO Ruby), wheat germ agglutinin conjugated with Alexa Fluor^*TM*^ 488 Conjugate (WGA), 4′,6-diamidino-2-phenylindole (DAPI), and Calcofluor White and FUN-1 dyes were purchased from Invitrogen (Thermo Fisher Scientific, Waltham, MA, United States). All chemical reagents and solvents employed in this study were of analytical grade.

### Strains and Cultural Conditions

*Candida albicans* SC5314 or ATCC 10231, *E. faecalis* 20033 or ATCC 29212, and the mixed-cultures of *C. albicans* SC5314 and *E. faecalis* 20033 were applied to investigate antimicrobial activity of luteolin. In addition, *C. albicans* SC5314 and *E. faecalis* 20033 were further employed for monomicrobial and polymicrobial biofilm formation and eradication assay. *C. albicans* strains were cultured from frozen stock in YPD medium and grown overnight at 37°C with shaking at 200 rpm, and TSB medium was used for culturing *E. faecalis* strains. Then, 1:1 mixed cultures of *C. albicans* SC5314 and *E. faecalis* 20033 were cultured in YPD/TSB (50:50) at 37°C with shaking at 200 rpm. From the overnight culture, 150 μl of aliquot were transferred to 3 ml of YPD, TSB, and YPD/TSB (50:50), respectively, and cultured at 37°C for 4–6 h to the mid-exponential phase of growth. The fungal and bacterial stocks were supplemented with a final concentration 25% (*v*/*v*) of glycerol and kept at −80°C.

### Determination of Minimal Inhibitory Concentration and Minimum Fungicidal/Bactericidal Concentration

The luteolin minimal inhibitory concentration (MIC) and minimum fungicidal/bactericidal concentration (MFC/MBC) values were measured according to previous studies (Schuurmans et al., 2009; Qian et al., 2020) with minor changes and evaluated as described by the European Committee on Antimicrobial Susceptibility Testing in a 96-well microplate (Costar, Corning, NY, United States). The mono- and dual-species overnight cultures were grown at 37°C until they reached mid-log phase for a total of five different cultures. *C. albicans* SC5314 or ATCC 10231, *E. faecalis* 20033 or ATCC 29212, and the mixed cultures of *C. albicans* SC5314 and *E. faecalis* 20033 strains grown in YPD, TSB, and YPD/TSB (50:50) media; 100 μl of the log-phase suspensions (10^6^ CFU ml^–1^) were transferred into each well of the 96-well plate. Furthermore, the luteolin was diluted to yield final concentrations between 2 and 512 μg ml^–1^. The final DMSO concentration of 5% (*v*/*v*) was used as the negative control. The microplate was then sealed with a parafilm and incubated at 37°C for 24 h. The MIC was determined as the lowest luteolin concentration at which no microbial growth was observed. Meanwhile, after culturing at 37°C for 24 h, the suspensions from each well were diluted and plated on YPD, TSA, or YPD/TSA (50:50) to count microbial cell numbers. The MFC/MBC is the lowest concentration of luteolin required to almost kill all fungal or bacterial cells present.

### Abiotic Surface Biofilms of *Candida albicans* and *Enterococcus faecalis* Single and Dual Species

Overnight cultures of *C. albicans* SC5314 and *E. faecalis* 20033 were further cultivated to a mid-logarithmic phase of growth for 6 h at 37°C. Subsequently, the standardized cell suspension (1 × 10^8^ CFU ml^–1^ in YPD and 1 × 10^8^ CFU ml^–1^ in TSB for *C. albicans* SC5314 and *E. faecalis* 20033, respectively) was prepared for single-species biofilms or 1:1 ratio of each suspension (1 × 10^8^ CFU ml^–1^ of *C. albicans* SC5314 plus 1 × 10^8^ CFU ml^–1^ of *E. faecalis* 20033) for mixed-species biofilms. The resulting cell suspensions of single and dual species were seeded in either each well of 96-well plate or on 14 mm × 14 mm glass coverslips in 24-well flat-bottom plate, respectively, and incubated in the absence of luteolin at 37°C. The plates were allowed to incubate over a total incubation period time of 24, 48, or 72 h with shaking at 50 rpm, followed by the renewing of media by adding a fresh sterile YPD, TSB, or YPD/TSB (50:50) medium for single or dual species, respectively. Subsequently, the glass coverslips were extensively rinsed with 10 mM PBS to remove non-adherent cells.

To evaluate biofilms by standardized cell suspensions of single and mixed species in the presence of luteolin, YPD, TSB, or YPD/TSB media were supplemented with conditioned luteolin (0, 1/4, 1/2, and 1 MIC) as indicated and incubated for 48 h at 37°C. The resulting biofilms were examined by estimating biofilm biomass using the crystal violet staining assay (CVSA) and optical microscope, by examining biofilm structure using field emission scanning electron microscope (FESEM) and confocal laser scanning microscope (CLSM), or by assessing matrix composition within biofilms using CLSM, according to previous reports (Alhede et al., 2012; Qian et al., 2020). All assays were performed independently and in triplicate.

### Adhesion Assay

The standardized cell suspensions of *C. albicans* SC5314 and *E. faecalis* 20033 were added to each well of the 24-well plate, where each well contains a glass coverslip and is supplemented with final concentrations of luteolin (0, 1/4, 1/2, and 1 MIC), followed by incubation at 37°C for 2 h with shaking at 50 rpm. Then, the supernatants were discarded from the plate and the glass coverslips were gently rinsed with 10 mM PBS to remove non-adherent cells. After treatment, the adhesion of cells on the glass coverslips was examined *via* CLSM. To determine the number of adherent cells, the biofilms were washed three times with 10 mM PBS to remove planktonic cells and then immersed in 10 ml of sterile water and sonicated at 55 kHz for 10 min. The resulting samples were diluted; plated onto YPD, TSA, or YPD/TSA plates; and incubated at 37°C for 24 h to examine the number of adherent cells. The number of adherent cells was determined as previously described (Qian et al., 2021b).

### Gatifloxacin Diffusion Within Biofilms of Luteolin-Mediated *Candida albicans* and *Enterococcus faecalis* Single and Dual Species

Antibiotic diffusion within biofilms was determined based on the intrinsic fluorescence of gatifloxacin by CLSM to evaluate the diffusion capability of antibiotics within biofilms formed in the presence of luteolin, according to previous studies (van der Waal et al., 2017; Jegal et al., 2019; Qian et al., 2021a). The standardized cell suspensions of *C. albicans* SC5314 and *E. faecalis* 20033 were cultured in each well of the 24-well plate and incubated in indicated doses of luteolin (0, 1/4, 1/2, and 1 MIC) at 37°C for 48 h with shaking at 50 rpm. The resulting biofilms formed on the glass coverslips were rinsed gently twice with 10 mM PBS (pH 7.4) and then exposed to a final concentration of 0.4 mg ml^–1^ gatifloxacin at 37°C for another 4 h. Next, to assess gatifloxacin diffusion within biofilms, the biofilm was stained with a final concentration of 2.5 μM of SYTO 9 and incubated for 20 min in the dark. The biofilm was then washed gently thrice with 10 mM PBS and measured using CLSM. The emission peak for gatifloxacin was recorded at 495 nm upon excitation at 291 nm.

### Effects of Luteolin on Biofilm Cells of *Candida albicans* and *Enterococcus faecalis* Single and Dual Species by Confocal Laser Scanning Microscope

The 48-h mature biofilms of single and dual species of *C. albicans* SC5314 and *E. faecalis* 20033 formed on the glass coverslips placed inside a 24-well plate were exposed to luteolin (0, 2, 4, and 8 MIC) at 37°C for another 4 h and then washed gently twice. Thereafter, the biofilms were mixed thoroughly with SYTO 9 and propidium iodide (PI) dyes and further incubated at 37°C for 20 min. Finally, stained live and dead cells inside the biofilm were visualized *via* CLSM. For determining metabolic activity of *C. albicans* SC5314 cells within single- or mixed-species biofilms, biofilm cells were also stained with FUN-1 (10 μM) with excitation/emission maxima of ∼490/617 and Calcofluor White (5 μM) for ∼380/475 nm at 37°C for 20 min, respectively. Finally, the images were captured by CLSM. To determine the population of viable biofilm cells, the resulting samples were diluted; plated onto YPD; TSA, or YPD/TSA plates; and incubated at 37°C for 24 h to evaluate the number of viable cells within biofilms (Qian et al., 2021b).

### Dispersal Effect of Luteolin on Mature Biofilms by *Candida albicans* and *Enterococcus faecalis* Single and Dual Species

To individual well of a 24-well plate containing a glass coverslip, 800 μl of overnight cultures were added. After 72 h of incubation at 37°C with shaking at 50 rpm, the biofilm was washed once with 10 mM PBS (pH 7.4) to wash away planktonic cells. Then, 72-h mature biofilms were exposed to luteolin with different concentrations (0, 4, 8, and 16 MIC) and incubated for another 5 h at 37°C. Finally, biofilms were examined by CVSA, visualized, and photographed using a light microscope or FESEM.

### Statistical Analyses

Statistical analyses were utilized to evaluate significant differences according to one-way analysis of variance (ANOVA), where Fisher’s least significant difference was performed. The results were graphed using GraphPad Prism version 5 (GraphPad Software, La Jolla, CA, United States).

## Results

### Minimal Inhibitory Concentration and Minimum Fungicidal/Bactericidal Concentration of Luteolin Against *Candida albicans* SC5314 and *Enterococcus faecalis* 20033 Single- and Dual-Species Cultures

Minimal inhibitory concentration and MFC/MBC of luteolin for the tested strains were examined, where the MIC and MFC/MBC values range between 32 and 128 μg ml^–1^ (Table 1). Single species of either *C. albicans* SC5314, *C. albicans* ATCC 10231, *E. faecalis* 20033, or *E. faecalis* ATCC 29212 (MIC = 32 μg ml^–1^; MFC/MBC = 64 μg ml^–1^) was more susceptible to luteolin than mixed cultures of *C. albicans* and *E. faecalis* (MIC = 64 μg ml^–1^; MFC/MBC = 128 μg ml^–1^). The results suggest that luteolin exhibited robust antimicrobial activity against *C. albicans* and *E. faecalis*.

**TABLE 1:** Minimum inhibitory non-contradiction (MIC), minimum fungicidal concentration (MFC) and minimum bactericidal concentrations (MBC) of luteolin against *C. albicans* and *E. faecalis*.

**Species**	**Strains**	**Luteolin (μg ml^–1^)**	
		**MIC**	**MFC/MBC**
*C. albicans*	SC5314	32	64
*C. albicans*	ATCC 10231	32	64
*E. faecalis*	20033	32	64
*E. faecalis*	ATCC 29212	32	64
Dual-species	SC5314, 20033	64	128

### Characterization of Single- and Dual-Species Biofilms of *Candida albicans* SC5314 and *Enterococcus faecalis* 20033

**FIGURE 1:**
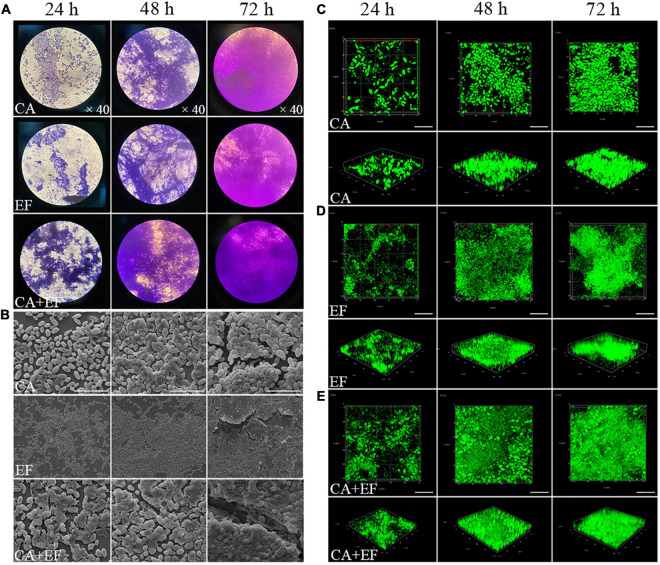
The biofilm structure of *C. albicans* SC5314 (CA) and *E. faecalis* 20033 (EF) single and dual species (CA + EF) at different cultivation times (24, 48, and 72 h) was assessed by crystal violet staining assay (CVSA) **(A)**, field emission scanning electron microscope (FESEM) **(B)** (magnification × 5,000), and confocal laser scanning microscope (CLSM) **(C–E)**. Scale bars represent 20 μm for CLSM.

As shown in Figure 1, either *C. albicans* SC5314 or *E. faecalis* 20033 was able to grow on a glass surface as a mono-species biofilm. The same performance was observed for *E. faecalis* 20033 and *C. albicans* SC5314 mixed species. There was a statistically significant difference between 24- and 48- or 72-h biomass of either single or mixed-species biofilms. Figure 1 displays representative micrographs of *C. albicans* SC5314 and *E. faecalis* 20033, in single- or dual-species biofilms after 72 h of incubation, captured by FESEM and CLSM. Figure 1 clearly demonstrates the integrity and depth of biofilms of both *C. albicans* SC5314 and *E. faecalis* 20033 single- and dual-species formed on a glass surface, which indicates the time-dependent manner in biofilm formation. Figures 1B,C also shows that *C. albicans* SC5314 and *E. faecalis* 20033 within mixed biofilms interacted intimately, and spherical cells of *C. albicans* immersed in a large amount of extracellular matrix inside biofilms. These results indicate that interstrain mutualism between *C. albicans* SC5314 and *E. faecalis* 20033 occurred, where the biofilm transition from initial surface colonization by solitary cells to a high-density segregated or mixed state was observed.

**FIGURE 2:**
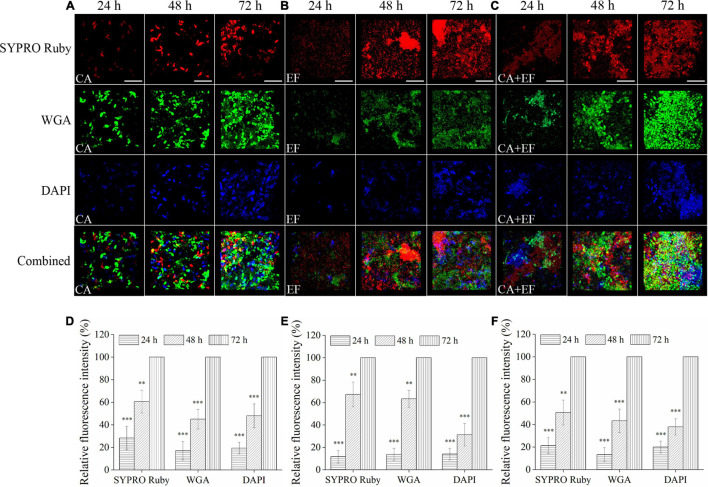
The matrix component levels within biofilms of *C. albicans* SC5314 (CA) **(A)** and *E. faecalis* 20033 (EF) **(B)** single and dual species (CA + EF) **(C)** at 24, 48, and 72 h were observed by confocal laser scanning microscope (CLSM) (scale bars of 20 μm). Extracellular proteins, polysaccharides, and eDNA inside biofilms were stained with SYPRO Ruby (5 μg/ml), wheat germ agglutinin conjugated with Alexa Fluor^*TM*^ 488 Conjugate (WGA) (5 μg/ml), and 4′,6-diamidino-2-phenylindole (5 μg/ml), respectively. The excitation/emission wavelengths were 450/610 nm for SYPRO Ruby, 495/519 nm for WGA, and 358/461 nm for 4′,6-diamidino-2-phenylindole (DAPI), respectively. The relative fluorescence intensity of matrix components within biofilms by *C. albicans* SC5314 **(D)**, *E. faecalis* 20033 **(E)**, and dual species **(F)** in different growth stage groups was calculated and plotted against those of the 72-h cultivation group using Image-Pro Plus 6.0 software, respectively. Scale bars represent the standard deviation (SD) (*n* = 3). ^∗∗^*p* < 0.01; ^∗∗∗^*p* < 0.001.

Confocal laser scanning microscope was also performed to investigate the matrix composition inside single- and dual-species biofilms of *C. albicans* SC5314 and *E. faecalis* 20033. For *C. albicans* SC5314 biofilms (Figure 2), the higher intensity of green fluorescence was measured compared with that of the red or blue fluorescence, and the green fluorescence increased from 24- to 72-h biofilms by ∼83% (*p* < 0.001), suggesting that the biofilm matrix was largely composed of polysaccharides and, to a lesser extent, proteins or eDNA. Similarly, for *E. faecalis* 20033 biofilm, the presence of abundant proteins labeled by red fluorescence was observed, and the red fluorescence enhanced from 24- to 72-h biofilms by ∼88% (*p* < 0.001), which indicated that extracellular proteins were the major structural components of the biofilm matrix. Additionally, for *C. albicans* SC5314 and *E. faecalis* 20033 mixed biofilms, more intensity of red and green fluorescence was observed, and the red and green fluorescence increased from 24- to 72-h biofilms by ∼78.7 and 86.7% (*p* < 0.001), respectively, suggesting that extracellular proteins and polysaccharides were the major structural components of the biofilm matrix. Furthermore, the images exhibited that the contents of proteins, polysaccharides, and eDNA increased consistently in a time-dependent manner.

### Inhibition of Luteolin Against Initial Cell-Surface Interaction

**FIGURE 3:**
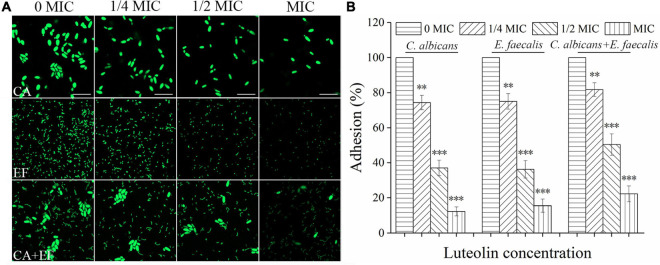
Luteolin exposure reduced cell adhesion of *C. albicans* SC5314 (CA) and *E. faecalis* 20033 (EF) single and dual species (CA + EF) on the glass surface. Cell adhesion was evaluated at final concentrations of luteolin ranging from 0 to 1 minimum inhibitory concentration (MIC) by confocal laser scanning microscope (CLSM) **(A)** (scale bars of 10 μm) and viable cell counts **(B)**. Error bars are expressed as means ± standard deviation (SD). ^∗^*p* < 0.05; ^∗∗^*p* < 0.01; ^∗∗∗^*p* < 0.001.

Figure 3 exhibits that luteolin reduced initial adherence of mono- and dual-species cells of *C. albicans* SC5314 and *E. faecalis* 20033 to the glass surface. For *C. albicans* SC5314 and *E. faecalis* 20033 single-species group treated with 1/2 MIC, the amount of the adhered cells was reduced by a ∼63–87.7% reduction, and a ∼63.8–84.5% reduction was observed at MIC-treated group, compared to the untreated group. Similarly, the amount of adhered cells in luteolin-treated samples of *C. albicans* SC5314 and *E. faecalis* 20033 mixed-species group was lower than their corresponding controls, resulting in a reduction from 49.7 to 77.8%. This difference was statistically significant between all 1/2- or MIC-treated group and untreated group (*p* < 0.001). However, the amount of adhered cells in luteolin-treated dual-species group was higher than that in luteolin-treated single species of *C. albicans* SC5314 and *E. faecalis* 20033 groups. Notably, CLSM images presented that almost all cells of *C. albicans* SC5314 and *E. faecalis* 20033 single-species group treated with 1/4- or 1/2-MIC luteolin were solitary on the glass surface. Conversely, more cell–cell adhesion of *C. albicans* SC5314 in the mixed-species group treated with 1/4- or 1/2-MIC luteolin was observed on the glass surface, which suggested that solitary cells can be reverted to sparse groups due to the disturbance event that removed most of the population.

### Luteolin Prevented the Biofilm Formation of *Candida albicans* SC5314 and *Enterococcus faecalis* 20033 Single and Dual Species

**FIGURE 4:**
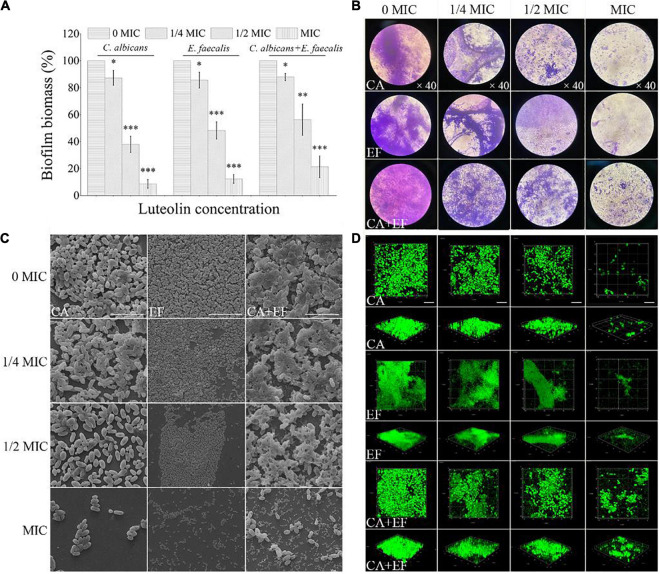
Inhibitory effects of various concentrations of luteolin on the biofilm formation of *C. albicans* SC5314 (CA) and *E. faecalis* 20033 (EF) single and dual species (CA + EF) were determined using crystal violet staining assay (CVSA) **(A)**, optical microscope **(B)**, field emission scanning electron microscope (FESEM) **(C)** (scale bars of 20 μm), and confocal laser scanning microscope (CLSM) **(D)** (scale bars of 20 μm). Values represent the means of triplicate measurements. Scale bars represent the standard deviation (SD) (*n* = 3). ^∗^*p* < 0.05; ^∗∗^*p* < 0.01; ^∗∗∗^*p* < 0.001.

The inhibitory effect of luteolin against biofilm formation of *C. albicans* SC5314 and *E. faecalis* 20033 single and dual species is displayed in Figure 4. Luteolin at concentrations equal to or higher than 16 and 32 μg ml^–1^ delivered significant decreases of the biofilm biomass of single-species biofilms, ranging from 62.0 and 51.7% to 91.4 and 87.7% for *C. albicans* SC5314 and *E. faecalis* 20033, respectively, compared to the corresponding negative control. For the mixed-species biofilms, exposure to luteolin at 16, 32, and 64 μg ml^–1^ elicited significant reductions in total biofilm biomass of 12.3, 43.7, and 78.7%, respectively. As shown in Figures 4B–D, biofilms in the luteolin-treated *C. albicans* SC5314 group appeared to be sparse and thinner attached to glass surface compared to the untreated group along with the rise of luteolin concentration. Moreover, after MIC luteolin treatment, the biofilm appeared cleaner and only a few aggregates of an adherent cluster of *C. albicans* SC5314 were apparent compared with the untreated group. Similarly, the weakening of luteolin-treated *E. faecalis* 20033 single-species biofilms appeared to be a dose-dependent inhibitory effect, as greater concentrations resulted in enhanced biofilm inhibition. Luteolin-treated biofilms at MIC appeared to be more scattered, and less microbial cell clusters on the glass surface were observed. Dual species of *C. albicans* SC5314 and *E. faecalis* 20033 without luteolin exposure formed three-dimensional biofilms with a smooth undulating profile of the biofilm surface, where *E. faecalis* 20033 tightly adhered to *C. albicans* SC5314. By contrast, when dual-species biofilms were exposed to luteolin, biofilms with a less dense and tight network were distributed non-homogeneously on the glass surface. Unlike in the control sample, in the MIC-treated samples of dual species, a mushroom-like structure of the biofilm disappeared, whereas only spotted cells could be observed.

### Luteolin Diminished the Amounts of Biofilm Matrix Components Inside Biofilms Formed by *Candida albicans* SC5314 and *Enterococcus faecalis* 20033 Single and Dual Species

**FIGURE 5:**
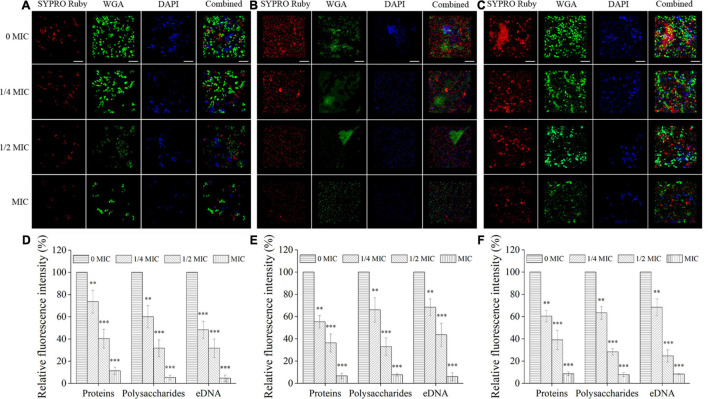
The effects of luteolin on the levels of the matrix component inside biofilms formed by *C. albicans* SC5314 **(A)**, *E. faecalis* 20033 **(B)**, and dual species **(C)** by confocal laser scanning microscope (CLSM). Extracellular proteins, polysaccharides, and eDNA were labeled with SYPRO Ruby, WGA, and 4′,6-diamidino-2-phenylindole (DAPI), respectively. Scale bars represent 20 μm. The relative fluorescence intensity of matrix components within biofilms by *C. albicans* SC5314 **(D)**, *E. faecalis* 20033 **(E)**, and dual species **(F)** in each exposure group was calculated and plotted against that in the untreated group using Image-Pro Plus 6.0 software, respectively. Scale bars represent the standard deviation (SD) (*n* = 3). ^∗∗^*p* < 0.01; ^∗∗∗^*p* < 0.001.

The biofilm matrices were assessed by CLSM in combination with three fluorescent dyes to evaluate the inhibitory effects of luteolin against the biofilm composition of *C. albicans* SC5314 and *E. faecalis* 20033 single and dual species. As illustrated in Figures 5A,D, for *C. albicans* SC5314 biofilms treated with sub-inhibitory concentrations of luteolin, compared with the untreated control, the levels of two biofilm matrix components (mainly polysaccharides and eDNA) inside the biofilms were reduced gradually with the increase of luteolin concentration, especially for the MIC-treated group, as evidenced by the decline in intensities of both green and blue fluorescences (100 and 100% vs. 5.3 ± 2.0% and 4.6 ± 2.8%, respectively; *p* < 0.001). Similarly, for *E. faecalis* 20033 biofilms, luteolin treatment reduced fluorescence intensities in both red and green fluorescences in a dose-dependent manner (Figures 5B,E), which indicated that luteolin at MIC reduced protein and polysaccharide levels within biofilms (100 and 100% vs. 6.6 ± 2.5% and 7.6 ± 1.1%, respectively; *p* < 0.001). Moreover, for biofilms of *C. albicans* SC5314 and *E. faecalis* 20033 mixed cultures, luteolin exposure diminished the levels of biofilm matrix components of mixed-species biofilms by synchronously downregulating the level of proteins, polysaccharides, and eDNA within biofilms (Figures 5C,F), as evidenced by a distinct reduction in the intensity of either red, green, or blue fluorescences in the MIC-treated group (100, 100, and 100% vs. 8.6 ± 1.5%, 7.8 ± 1.7%, and 8.3 ± 0.5%, respectively; *p* < 0.001).

### Antibiotic Diffusion Increased Within Luteolin-Treated Biofilms by *Candida albicans* SC5314 and *Enterococcus faecalis* 20033 Single and Dual Species

**FIGURE 6:**
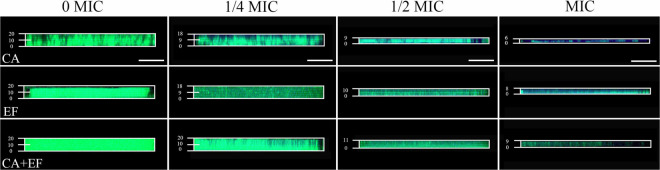
Examination of the diffusion of antibiotics within *C. albicans* SC5314 (CA) and *E. faecalis* 20033 (EF) single and dual species (CA + EF) biofilms formed in the presence of luteolin using gatifloxacin as a model by confocal laser scanning microscope (CLSM). Biofilms were examined with SYTO 9 (green) and the intrinsic fluorescence of gatifloxacin (blue). Scale bars represent 20 μm.

The penetration of antibiotics into the biofilm of single and dual species exposed to luteolin was investigated using gatifloxacin as a model. As shown in Figure 6, there was little visible blue fluorescence within biofilms of *C. albicans* SC5314 and *E. faecalis* 20033 single and dual species in the untreated control group, indicating that biofilms without exposure to luteolin demonstrated a high level of resistance for gatifloxacin. The results demonstrated that the addition of luteolin obviously increased the content of blue fluorescence in biofilms of *C. albicans* SC5314 and *E. faecalis* 20033 single species. The total blue fluorescence intensity within biofilms was positively correlated with the concentration of luteolin. In the presence of 1/4 MIC or MIC of luteolin, plenty of blue fluorescences were observed within biofilms of *C. albicans* SC5314 single species compared to the untreated group, exhibiting enhanced susceptibility to luteolin. Similarly, in *E. faecalis* 20033 single-species samples treated with 1/4 MIC or greater concentrations, the results showed that diffusion of gatifloxacin inside treated biofilms was significant with numerous blue-fluorescence compared with the untreated control group. By contrast, for the 1/4 MIC-treated dual-species biofilms, few blue fluorescences were visible in the external rim of the biofilm, leaving very few fluorescences in the interior of the biofilm. This result suggested that mixed biofilms treated with luteolin were generally less susceptible against gatifloxacin diffusion than luteolin-treated single-species biofilms.

### Luteolin Displayed the Killing Effect on Biofilm-Related Cells of *Candida albicans* SC5314 and *Enterococcus faecalis* 20033 Single and Dual Species

**FIGURE 7:**
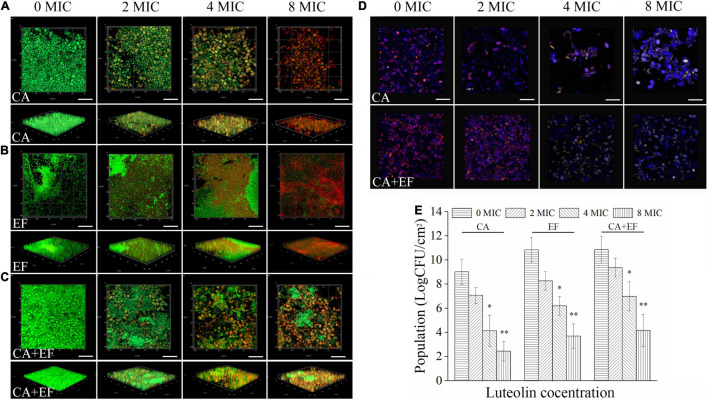
Assessment of the killing effect of luteolin on biofilm cells of *C. albicans* SC5314 (CA) **(A)** and *E. faecalis* 20033 (EF) **(B)** single and dual species (CA + EF) **(C)** *via* confocal laser scanning microscope (CLSM). Representative CLSM photographs of plane and three-dimensional luteolin-treated biofilms were displayed. Viable and non-viable biofilm cells were examined by SYTO 9 (green) and PI (red), respectively. Metabolic activity assay of *C. albicans* SC5314 cells within biofilms by luteolin-treated single and dual species **(D)** (scale bars of 20 μm). Red fluorescences represent the viable channel of fungal cells; yellow-green fluorescences stand for the non-viable channel of fungal cells. The number of viable cells within biofilms by luteolin-mediated *C. albicans* SC5314 and *E. faecalis* 20033 single and dual species was evaluated by the viable cell counting method **(E)**. ^∗^*p* < 0.05; ^∗∗^*p* < 0.01.

The killing effect of luteolin on biofilm cells of single and dual species was verified using CLSM equipped with SYTO 9 and PI dyes. As exhibited in Figures 7A–C, pronounced higher levels of green fluorescence within biofilms were observed in the *C. albicans* SC5314 and *E. faecalis* 20033 single- and dual-species untreated samples when compared with the luteolin-treated groups, suggesting that most of biofilm cells remained viable. After treatment with luteolin, red fluorescence within biofilms was significantly increased, and the effect of luteolin on the increase of red fluorescence was dose dependent. Moreover, treatment of *C. albicans* SC5314 and *E. faecalis* 20033 single- and dual-species biofilms with luteolin at four MIC or eight MIC reduced the abundance of green fluorescence significantly and remarkably increased the intensity of red fluorescence accordingly, compared with the negative control. Especially, eight MIC luteolin demonstrated more effective against single-species biofilm cells than cells within mixed-species biofilms, where green fluorescence remained in relatively small amounts; most of viable cells of mixed species treated with high-concentration luteolin were *E. faecalis* 20033, indicating that luteolin exerted a preferential effect on *C. albicans* SC5314 within dual-species biofilms. Additionally, the log10 reduction in viable cells within biofilms was 6.6 ± 1.0, 7.1 ± 1.6, and 6.6 ± 1.5 after addition of eight MIC of luteolin to *C. albicans* SC5314, *E. faecalis* 20033, and dual-species biofilms, respectively (Figure 7E), suggesting that luteolin showed a significant effect on the viability of *C. albicans* SC5314 and *E. faecalis* 20033 cells within biofilms. Similarly, the transition from red to yellow-green fluorescence of biofilm cells occurred in the treated samples of *C. albicans* SC5314 single or dual species, indicating that biofilm cells of *C. albicans* SC5314 lost metabolic activity upon treatment with high-concentration luteolin (Figure 7D), which supported the findings of CLSM in combination with live/dead staining with SYTO 9 and PI.

### Luteolin Eradicated Efficiently Preformed Biofilms Formed by *Candida albicans* SC5314 and *Enterococcus faecalis* 20033 Single and Dual Species

**FIGURE 8:**
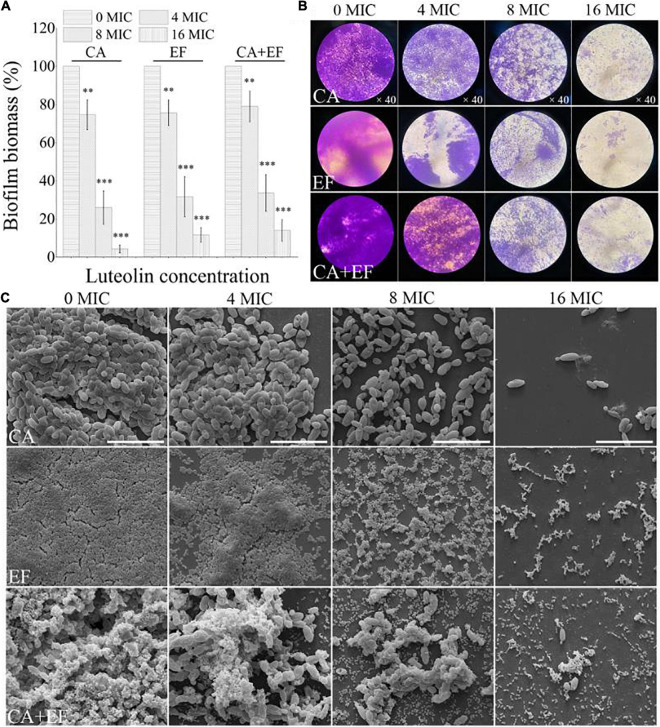
Dispersal effects of various concentrations of luteolin on 72-h-old mature biofilms of *C. albicans* SC5314 (CA) and *E. faecalis* 20033 (EF) single and dual species (CA + EF) were assessed using crystal violet staining assay (CVSA) **(A)**, optical microscope **(B)**, and field emission scanning electron microscope (FESEM) **(C)** (scale bars of 20 μm). Values represent the mean of triplicate measurements. Scale bars represent the standard deviation (SD) (*n* = 3). ^∗^*p* < 0.05; ^∗∗^*p* < 0.01; ^∗∗∗^*p* < 0.001.

The efficacy of luteolin to remove 72-h-old biofilms by the single and dual species of *C. albicans* SC5314 and *E. faecalis* 20033 was evaluated using CVSA and FESEM. As shown in Figure 8, the outcome of the application of luteolin to the mature biofilms of single and dual species of *C. albicans* SC5314 and *E. faecalis* 20033 was highly similar between treatments, showing that luteolin significantly dispersed the 72-h-old biofilms. For *C. albicans* SC5314 and *E. faecalis* 20033 single-species biofilms, biofilm biomass assessment using CVSA exhibited a 74.0 and 68.4% reduction in the samples, respectively, when treated with 8 MIC compared to the corresponding untreated samples. Furthermore, the biofilms of *C. albicans* SC5314 and *E. faecalis* 20033 single species exposed to luteolin at 16 MIC were almost completely removed. Similarly, the luteolin treatment at 8 MIC or 16 MIC had an obvious removal effect on the biofilm biomass of established biofilms of dual species. Strikingly, FESEM images from the luteolin treatments correlated better with the CVSA examination. The dense and compact biofilms of *C. albicans* SC5314 and *E. faecalis* 20033 single and dual species disappeared, and only few and scattered tiny aggregates on the glass surface were observed when the samples were treated with 16 MIC.

## Discussion

*Candida albicans* and *E. faecalis* are often co-isolated together from complex biofilm communities, resulting in the challenge of chronic, recalcitrant infections in large part because of their high ability to form biofilms, which reduce the effects of conventional antimicrobial agents (Hermann et al., 1999; Mayr et al., 2014). Therefore, exploiting a safe and effective agent for inhibition of biofilm formation as well as mature biofilm removal of *C. albicans* and *E. faecalis* single and dual species is of great value. Nowadays, the exploration of plant-derived natural compounds has shown great promise, as significant sources for novel, natural antimicrobial agents particularly for the treatment of intractable infections caused by biofilms (Lu et al., 2019). Luteolin has demonstrated high-efficacy and low-toxicity effects in multiple *in vitro* and *in vivo* studies, showing a promising potential as a clinical candidate with the aim of preventing coronary arterial spasm (Nabavi et al., 2015; Li et al., 2019; Swaminathan et al., 2019).

In this study, we assessed mainly the efficacy of luteolin on the inhibition of biofilm formation as well as mature-biofilm removal of *C. albicans* SC5314 and *E. faecalis* 20033 single and dual species and evaluated the sensitivity of biofilm-associated cells to luteolin treatment. Before assessing the effect of luteolin on the biofilm of single culture and mixed cultures, MIC and MFC/MBC were determined for luteolin using a twofold serial dilution assay. MIC and MFC/MBC values of luteolin were 32 and 64 μg ml^–1^ against *C. albicans* SC5314, *C. albicans* ATCC 10231, *E. faecalis* 20033, or *E. faecalis* ATCC 29212 single culture and 64 and 128 μg ml^–1^ against mixed cultures, respectively, which exhibits strong antimicrobial activity against single culture and mixed cultures of *C. albicans* and *E. faecalis*. Similar reports displayed that the MIC of luteolin against *S. aureus* (Joung et al., 2016), *E. coli*, *Enterobacter cloacae*, and *Trueperella pyogenes* was found to be 62.5–128 μg ml^–1^, respectively (Guo et al., 2020; Qian et al., 2021a).

The existing evidences indicate that the co-existence of *C. albicans* and *E. faecalis* was found in tongue mucosal infections (Dahlen et al., 2012), sputum, sepsis (Hermann et al., 1999), and root canal infections (Abusrewil et al., 2020). However, there were certain differences in the interactive profiles of dual-species biofilm communities made up of *C. albicans* and *E. faecalis* on a variety of substrates. For instance, Krishnamoorthy et al. (2020) reported that *C. albicans* (SC5314 and BF1) and *E. faecalis* (OG1RF and P52S) acted mutually on an organotypic oral mucosal model, where the dual-species biofilms resulted in profound surface erosion compared to either species alone. Conversely, Graham et al. (2017) exhibited that *E. faecalis* OG1RF was antagonistic to biofilm formation of *C. albicans* SC5314 on a polystyrene substrate. Therefore, to evaluate antibiofilm effects of luteolin on biofilms by *C. albicans* SC5314, *E. faecalis* 20033, and mixed species, the biofilm formation of single and mixed cultures was evaluated on a glass surface. Our results demonstrated that *C. albicans* SC5314 and *E. faecalis* 20033 formed a synergistic partnership on the glass surface. The potential mechanisms might be attributed to the adhesin–glass interactions mediated by *E. faecalis* 20033, resulting in more clumps or coaggregation between *C. albicans* SC5314 and *E. faecalis* 20033. Moreover, CLSM results further showed that the biofilm matrix of *E. faecalis* 20033 and mixed-species biofilms formed on a glass surface appeared to consist mainly of extracellular proteins and polysaccharides.

The formation of a biofilm initiates the attachment of microbial cells to the substrate surface, followed by the coaggregation and proliferation of microbial cells associated with the formation of multilayer cell clusters (Stoodley et al., 2002). Herein, the efficacy of luteolin against biofilms of *C. albicans* SC5314 and *E. faecalis* 20033 single and mixed species was evaluated by CVSA, optical microscope, FESEM, and CLSM. Our results showed that compared with the untreated control, luteolin at 16 μg ml^–1^ could inhibit the adhesion of *C. albicans* SC5314 and *E. faecalis* 20033 single culture by 63% and by 63.8%, respectively, as well as the adhesion of mixed cultures by 49.7% at 32 μg/ml. Moreover, luteolin at 32 and 64 μg ml^–1^ could remarkably reduce the biofilm biomass of *C. albicans* SC5314, *E. faecalis* 20033, and mixed species by 91.4, 87.7, and 78.7%, respectively. In comparison, Shin and Eom (2019) reported that compared with the untreated control, zerumbone at 64 μg ml^–1^ restrained the biofilm biomass of *C. albicans* ATCC 14053 and *S. aureus* ATCC 29213 by 73.3 and 80.1%, respectively, and at 128 μg ml^–1^ effectively inhibited the biofilm biomass of mixed species by 98.9%. Together, these findings could signify that luteolin was effective as a potential potent antibiofilm agent in the control of both the initial and intermediate stages of biofilm formation of *C. albicans*, *E. faecalis*, and mixed species.

Next, we further explored the content of biofilm matrix components within biofilms formed by *C. albicans* SC5314, *E. faecalis* 20033, and mixed cultures in the presence of luteolin. Our results indicate that treatment of luteolin decreased the levels of matrix components of *C. albicans* SC5314 by synchronously downregulating the level of exopolysaccharides, proteins, and eDNA within biofilms, whereas luteolin inhibited biofilm biomass of *E. faecalis* 20033 and mixed cultures predominantly by diminishing the yield of exopolysaccharides and proteins inside biofilms in a dose-dependent manner. Furthermore, our results also indicate that treatment of luteolin improved the sensitivity of biofilms of *C. albicans* SC5314, *E. faecalis* 20033, and mixed cultures to antibiotics, thereby enabling these biofilm cells more vulnerable to antimicrobial insults. This phenomenon was consistent with the fact that the biofilm matrix component within biofilms has long been considered the major contributor to biofilm antimicrobial tolerance (Karygianni et al., 2020). These findings indicate that in addition to suppression of cell adhesion and attachment, the antibiofilm effects of luteolin also relied on interrupting the generation of biofilm matrix components, thereby blocking biofilm formation and increasing antimicrobial treatment.

In particular, biofilms are often refractory in the response to antimicrobial treatment, resulting in a multifactorial resistance phenomenon (Karygianni et al., 2020; Zhang et al., 2020). The current results confirm that luteolin, at concentrations of 128 μg ml^–1^, was able to kill/damage microbial cells within preformed biofilms of either *C. albicans* SC5314 or *E. faecalis* 20033, as well as at 256 μg ml^–1^ damage mature biofilm cells of mixed cultures. Likewise, previous reports showed that ciprofloxacin, tetracycline, and ampicillin were found to effectively penetrate and diffuse through bacterial biofilms (Verderosa et al., 2019). Unfortunately, many of these antibiotics are ineffective against mature biofilms (Verderosa et al., 2019). Interestingly, the present study indicates that luteolin exerted a powerful action in dispersing/eradicating mature biofilms formed by *C. albicans* SC5314, *E. faecalis* 20033, and mixed cultures, suggesting that targeting biofilm matrix components could offer a powerful tool against biofilm-associated infections caused by bacteria and/or fungi. These results display that luteolin might be a promising candidate against well-established biofilms of *C. albicans* SC5314 and *E. faecalis* 20033 single and dual species. However, further investigations involving the development of luteolin in conjunction with conventional antibiotics should be warranted to obtain potential antimicrobial therapies against recalcitrant fungal and bacterial mixed infections.

## Data Availability Statement

The original contributions presented in the study are included in the article/supplementary material, further inquiries can be directed to the corresponding author.

## Author Contributions

WQ: conceptualization and methodology. YF and WW: acquisition of the data. TW and QZ: analysis and interpretation of the data. YF: drafting of the manuscript. All authors critical revision of the manuscript for important intellectual content.

## Conflict of Interest

The authors declare that the research was conducted in the absence of any commercial or financial relationships that could be construed as a potential conflict of interest.

## Publisher’s Note

All claims expressed in this article are solely those of the authors and do not necessarily represent those of their affiliated organizations, or those of the publisher, the editors and the reviewers. Any product that may be evaluated in this article, or claim that may be made by its manufacturer, is not guaranteed or endorsed by the publisher.
